# Heterogeneity in Racial and Ethnic Disparities in COVID-19 Severity Among Pediatric Inpatients in a National Healthcare Database

**DOI:** 10.1093/jpids/piaf092

**Published:** 2025-10-08

**Authors:** David Watson, Alicen B Spaulding, Laura Norton

**Affiliations:** Division of Clinical Trials and Biostatistics, Department of Quantitative Health Sciences, Mayo Clinic, Rochester, Minnesota, United States; Research Institute, Children’s Minnesota, Minneapolis, Minnesota, United States; Research Institute, Children’s Minnesota, Minneapolis, Minnesota, United States; Division of Infectious Diseases, Department of Pediatrics, University of Minnesota Medical School, Minneapolis, Minnesota, United States

**Keywords:** effect modification, health equity, hospitalizations, ventilator

## Abstract

**Background:**

The COVID-19 pandemic magnified longstanding racial and ethnic disparities in pediatric health, but it is unclear which populations experienced the largest disparities. Our objective was to determine whether disparities in COVID-19 severity differed with respect to patient factors analyzed as effect modifiers.

**Methods:**

Using data from the Premier Healthcare Database, this retrospective cohort study included encounters among inpatients < 19 years old from April 2020 through September 2022 in the USA with a COVID-19 diagnosis. Outcomes of COVID-19 severity were intensive care unit (ICU) admission and ventilator use. Comparisons between Black and White patients and Hispanic and White patients were adjusted for confounders using propensity score weights, reported as risk differences (RDs) in percentage points, and tested for heterogeneity (interaction *P*) across subgroups of effect modifiers such as complex chronic conditions (CCCs) and insurance status.

**Results:**

Of 8947 pediatric inpatients with primary COVID-19 diagnosis, 3858 were White, 2153 were Black, and 2936 were Hispanic. Among children with a CCC, 14.3% of Black inpatients required a ventilator compared to 9.8% of White inpatients; among children without a CCC, 3.2% of both Black and White inpatients required a ventilator (RDs 4.5 vs 0.0; interaction *P* = .013). Comparisons of Hispanic and White inpatients showed a similar trend in ventilator use, with larger disparities among inpatients with CCCs and no difference among those without CCCs (RDs 2.7 vs −0.7; interaction *P*=.031). Among children with government insurance, 25.9% of Black inpatients were admitted to the ICU compared to 20.8% of White inpatients; among children with private insurance, Black and White inpatients had comparable ICU admission rates of 20.0% and 21.4%, respectively (RDs 5.1 vs −1.4; interaction *P* = .025).

**Conclusions:**

Among hospitalized children, racial and ethnic disparities in COVID-19 severity were largest for those with CCCs or government insurance. These results can help identify target populations for interventions to reduce inequity.

## INTRODUCTION

The COVID-19 pandemic magnified longstanding racial and ethnic disparities in pediatric health, driven by structural and social determinants of health, but greater understanding is needed regarding which factors drive these disparities among children. Prior studies have shown that children from racial and ethnic minority groups are at a higher risk for severe COVID-19 compared with non-Hispanic White children^1-3^ and that chronic medical conditions are associated with severe COVID-19 illness in children.^4^^,^^5^

Understanding which specific pediatric populations experience the greatest disparities in COVID-19 severity can help guide intervention strategies and inform equity-based approaches to clinical practice.^6^ To extend our understanding of disparities beyond a monolithic view of race, analysis of effect modification can assess heterogeneity in disparities observed within specific subgroups of patients. This approach segments the population to assess whether racial disparities within subgroups vary. For example, we previously reported variability in racial disparities among pediatric COVID-19 patients based on whether patients had a complex comorbidity. Among patients with a complex comorbidity, disparities in hospitalization rates were much larger than disparities among patients without such conditions.^7^ In the current study, we seek to expand upon our previous findings using a different national healthcare database to explore additional effect modifiers for higher severity outcomes in disparities of COVID-19 burden among hospitalized pediatric patients.

## METHODS

### Study Setting and Population

This study used a retrospective cohort extracted from the Premier Healthcare Database (Premier), which contains information on inpatient encounters from geographically diverse hospitals across the USA.^8^^,^^9^ Since 2012, Premier has included more than 11 million inpatient encounters per year. Outpatient and emergency department encounters are included from clinics within the same health system as participating hospitals, and unique patients can be tracked within each health system. Premier includes patient-, service-, and provider-level information, including International Classification of Diseases, Tenth Revision (ICD-10) codes, and charge master data that provide descriptions of diagnoses and services provided in the hospital. Data are considered deidentified in accordance with the Privacy Rule of the Health Insurance Portability and Accountability Act, and the Institutional Review Board at Children’s Minnesota determined that this study was not human subjects research.

The initial data query from Premier included all encounters with ICD-10 code U07.1 for a COVID-19 diagnosis occurring from January 2020 through September 2022 among patients less than 19 years old. For patients meeting these criteria, all patient encounters from the time frame were included in the extract. From this initial query, the cohort for this study included inpatient admissions with primary diagnosis of COVID-19 between April 2020 and September 2022. Additional exclusion criteria were patients with diagnosis of multisystem inflammatory syndrome in children (ie, ICD-10 code M35.81), nonemergency/nonurgent admissions (ie, elective, newborn, trauma center, or unknown), and patients with unknown sex, race, or ethnicity. Only the first inpatient encounter with a primary diagnosis of COVID-19 was included.

### Data Definitions

Outcome measures of COVID-19 severity during an encounter were intensive care unit (ICU) admission or ventilator use as identified from charge master data. Race and ethnicity were determined from standardized billing forms (ie, UB-04); race was reported as White, Black, Asian, other, or unknown; and ethnicity was reported as Hispanic, not Hispanic, or unknown. For this study, race and ethnicity were categorized into three groups: the reference group of non-Hispanic White, Hispanic (of any race), and non-Hispanic Black (henceforth, the “non-Hispanic” descriptor will be implicit), similar to categorizations used in prior COVID-19 severity studies.^2^^,^^3^ Sample sizes for the remaining racial groups were insufficiently powered to detect effect modification.

Potential effect modifiers included any complex comorbidities (vs none), patient’s insurance type (private or government, ie, Medicaid), age group (<1, 1-5, 6-11, and 12-18 years), and obesity. Having a complex comorbidity was defined as having at least one complex chronic condition (CCC) outlined by Feudtner et al.^10^ The rationale for selecting these characteristics as effect modifiers was to identify groups with the largest disparities with the ultimate goal of improving equity within these groups. One constraint on selecting effect modifiers (and comparison groups) was having a reasonable number of patients in each subgroup in order to estimate interaction effects, which require much larger sample sizes than main effects.^11^

Effect modifiers were considered confounders when not explicitly analyzed for effect modification (eg, when assessing complex comorbidities as an effect modifier, insurance type, age group, and obesity were considered confounders). Calendar quarter was considered a confounding factor to account for changes over the course of the pandemic. Additional hospital-level confounders included US region, rural versus urban location, hospital bed size, and whether the hospital was a teaching institution. Urban status was determined using the US Census definition of an urban area, and teaching institutions had an affiliation with a medical school. Additional confounders were 12 underlying medical conditions, including obesity, that were associated with COVID-19 severity among children as reported in a prior study^4^ that defined these conditions via a combination of the Chronic Condition Indicator^12^ and Clinical Classifications Software Refined.^13^ In addition to these underlying medical conditions, the 11 specific groupings of CCCs were considered confounders (eg, cardiovascular and respiratory). Underlying medical conditions and CCCs are two distinct techniques to identify certain—and perhaps overlapping—conditions based on ICD-10 codes; the former was an ad hoc method based on classifications from the Healthcare Cost & Utilization Project and the latter follows a long line of work on pediatric conditions that have higher mortality risk and healthcare utilization.^14^ Both types of conditions were identified via ICD-10 codes from the inpatient admission and/or any prior encounter in the data extract.

Vaccination status was one known confounding factor that was not available within Premier. To address this limitation, we performed a sensitivity analysis looking specifically at whether patients were eligible for vaccines as an effect modifier. Details on the rationale and definitions of vaccine eligibility are provided in the online supplementary material.

### Weighting Design and Statistical Methods

To adjust for confounding, we used propensity score analysis. Although developed for assessing causal effects of treatments,^15^ propensity score methods have been recommended for use in assessing racial disparities because they ensure that comparison groups are comparable with respect to observed confounding factors.^16^^,^^17^ Moreover, overlap weighting—the specific method we used—has been recommended for analysis of racial disparities and offers appealing properties for assessing effect modification.^18^^,^^19^ For this analysis, the propensity score and overlap weights were estimated within each level of the effect modifier^20^ using logistic regression with a binary outcome of race or ethnic group. After applying overlap weights, a mathematical property of this approach guarantees that all the confounding factors included in the propensity score are on average the same between comparison groups.^21^ This property of exact balance is the main benefit of this method and is demonstrated with balance diagnostics of standardized average differences before and after weighting.^22^ In contrast, other propensity score methods (eg, matching or inverse probability weighting) do not guarantee balance and require assessing balance diagnostics before moving on to comparisons of outcomes.^23^ In the context of analyzing effect modification, this property is useful because we do not have to assess balance for every racial or ethnic comparison within each subgroup of effect modifiers. Overlap weighting works best when comparing two groups, so weights were calculated separately for comparisons of Black and White patients and Hispanic and White patients (ie, outcomes may differ slightly for the White population across comparisons).

Outcomes of COVID-19 severity were summarized within each level of the effect modifiers for comparisons of Black patients to White patients and Hispanic patients to White patients. Both unweighted and weighted comparisons used linear regression with robust standard errors, and results on disparities are reported as risk differences (RDs) in percentage points with 95% confidence intervals (CIs). Robust standard errors account for non-normality of the outcomes, heteroscedasticity of residual variance, and overlap weights. The statistical test of the interaction between the effect modifier and racial and ethnic group is a formal test of effect modification with null hypothesis that all RDs are the same across subgroups of an effect modifier, so a statistically significant test at the 5% level suggests disparities vary across levels of the effect modifier. Analyses were performed using R (version 4.1.3) and SAS Enterprise Guide software (version 7.12).

## RESULTS

### Study Population

After applying inclusion and exclusion criteria to the initial data extract, the cohort had 8947 pediatric inpatients with a primary diagnosis of COVID-19 (Figure S1), of which 3858 were White, 2936 were Hispanic, and 2153 were Black. Racial and ethnic groups differed with respect to demographics, hospital characteristics, and medical conditions (Table 1). On average, Black patients were older than White patients, and Black and Hispanic patients were more likely to have government insurance than White patients. Among underlying conditions and CCCs, obesity, type 2 diabetes, asthma, and hematologic and immunologic conditions were more prevalent among Black patients than White patients.

**Table 1:** Patient Characteristics by Racial and Ethnic Groups

**Characteristic**	**Total** **(*n* = 8947)**	**White** **(*n* = 3858)**	**Hispanic** **(*n* = 2936)**	**Black** **(*n* = 2153)**	***P*-value**
Age, mean (SD)	6.7 (6.8)	6.5 (6.8)	6.3 (6.8)	7.7 (6.8)	<.001
Age, median (IQR)	4 (0, 14)	3 (0, 14)	3 (0, 13)	7 (1, 15)	<.001
Age group, *n* (%)					<.001
<1 year	2793 (31.2)	1247 (32.3)	1010 (34.4)	536 (24.9)	
1-5 years	2108 (23.6)	952 (24.7)	687 (23.4)	469 (21.8)	
6-11 years	1158 (12.9)	453 (11.7)	371 (12.6)	334 (15.5)	
12-18 years	2888 (32.3)	1206 (31.3)	868 (29.6)	814 (37.8)	
Female, *n* (%)	4170 (46.6)	1817 (47.1)	1343 (45.7)	1010 (46.9)	.513
Insurance type, *n* (%)					<.001
Private	2574 (28.8)	1696 (44.0)	483 (16.5)	395 (18.3)	
Government	5768 (64.5)	1913 (49.6)	2186 (74.5)	1669 (77.5)	
Other	605 (6.8)	249 (6.5)	267 (9.1)	89 (4.1)	
Admission era^a^, *n* (%)					<.001
Initial variants	2039 (22.8)	659 (17.1)	826 (28.1)	554 (25.7)	
Delta variant	2383 (26.6)	1054 (27.3)	739 (25.2)	590 (27.4)	
Omicron variant	4525 (50.6)	2145 (55.6)	1371 (46.7)	1009 (46.9)	
Census Region, *n* (%)					<.001
Midwest	1596 (17.8)	926 (24.0)	268 (9.1)	402 (18.7)	
Northeast	1140 (12.7)	334 (8.7)	493 (16.8)	313 (14.5)	
South	4963 (55.5)	2019 (52.3)	1619 (55.1)	1325 (61.5)	
West	1248 (13.9)	579 (15.0)	556 (18.9)	113 (5.2)	
Rural	483 (5.4)	239 (6.2)	105 (3.6)	139 (6.5)	<.001
Bed size, *n* (%)					<.001
<200	1388 (15.5)	466 (12.1)	544 (18.5)	378 (17.6)	
200-299	1273 (14.2)	447 (11.6)	512 (17.4)	314 (14.6)	
300-399	824 (9.2)	412 (10.7)	243 (8.3)	169 (7.8)	
400-499	929 (10.4)	522 (13.5)	252 (8.6)	155 (7.2)	
≥500	4533 (50.7)	2011 (52.1)	1385 (47.2)	1137 (52.8)	
Teaching institution, *n* (%)	6252 (69.9)	2767 (71.7)	1879 (64.0)	1606 (74.6)	<.001
Underlying cond.^b^, *n* (%)					
Diabetes mellitus, type 1	125 (1.4)	42 (1.1)	42 (1.4)	41 (1.9)	.035
Obesity	1280 (14.3)	463 (12.0)	455 (15.5)	362 (16.8)	<.001
Cardiac and circulatory congenital anomalies	708 (7.9)	310 (8.0)	246 (8.4)	152 (7.1)	.212
Epilepsy; convulsions	675 (7.5)	291 (7.5)	218 (7.4)	166 (7.7)	.930
Trauma- and stressor-related disorders	113 (1.3)	57 (1.5)	32 (1.1)	24 (1.1)	.285
Neurodevelopmental disorders	887 (9.9)	450 (11.7)	226 (7.7)	211 (9.8)	<.001
Diabetes mellitus, type 2	194 (2.2)	36 (0.9)	62 (2.1)	96 (4.5)	<.001
Depressive disorders	327 (3.7)	174 (4.5)	91 (3.1)	62 (2.9)	<.001
Essential hypertension	425 (4.8)	154 (4.0)	122 (4.2)	149 (6.9)	<.001
Anxiety- and fear-related disorders	568 (6.3)	315 (8.2)	154 (5.2)	99 (4.6)	<.001
Asthma	1821 (20.4)	690 (17.9)	539 (18.4)	592 (27.5)	<.001
Sleep-wake disorders	514 (5.7)	198 (5.1)	149 (5.1)	167 (7.8)	<.001
Complex chronic cond.^b^, *n* (%)					
Any	3130 (35.0)	1223 (31.7)	964 (32.8)	943 (43.8)	<.001
Neurologic or neuromuscular	822 (9.2)	342 (8.9)	279 (9.5)	201 (9.3)	.641
Cardiovascular	943 (10.5)	397 (10.3)	294 (10.0)	252 (11.7)	.122
Respiratory	492 (5.5)	210 (5.4)	167 (5.7)	115 (5.3)	.849
Renal	273 (3.1)	114 (3.0)	89 (3.0)	70 (3.3)	.812
Gastrointestinal	361 (4.0)	142 (3.7)	131 (4.5)	88 (4.1)	.266
Hematologic or immunologic	804 (9.0)	216 (5.6)	197 (6.7)	391 (18.2)	<.001
Metabolic	935 (10.5)	369 (9.6)	285 (9.7)	281 (13.1)	<.001
Congenital or genetic	582 (6.5)	273 (7.1)	184 (6.3)	125 (5.8)	.131
Malignancy	251 (2.8)	115 (3.0)	95 (3.2)	41 (1.9)	.012
Neonatal	334 (3.7)	139 (3.6)	101 (3.4)	94 (4.4)	.194
Transplant	79 (0.9)	30 (0.8)	29 (1.0)	20 (0.9)	.635
Number of cond., mean (SD)	0.66 (1.15)	0.61 (1.13)	0.63 (1.15)	0.78 (1.18)	<.001

Abbreviations: cond., condition; SD, standard deviation; Q1, first quartile; Q3, third quartile.

a For conciseness, eras of the COVID-19 pandemic are used instead of every quarter; the COVID-19 pandemic was divided into three distinct eras based on the dominant variants in the USA: initial variants (eg, alpha, beta, gamma) (April 2022, the start of the study period, through May 2021), Delta variant (June 2021 through November 2021), and Omicron variant (December 2021 through September 2022, the end of the study period).

b Underlying conditions and complex chronic conditions are not mutually exclusive.

**Figure 1:**
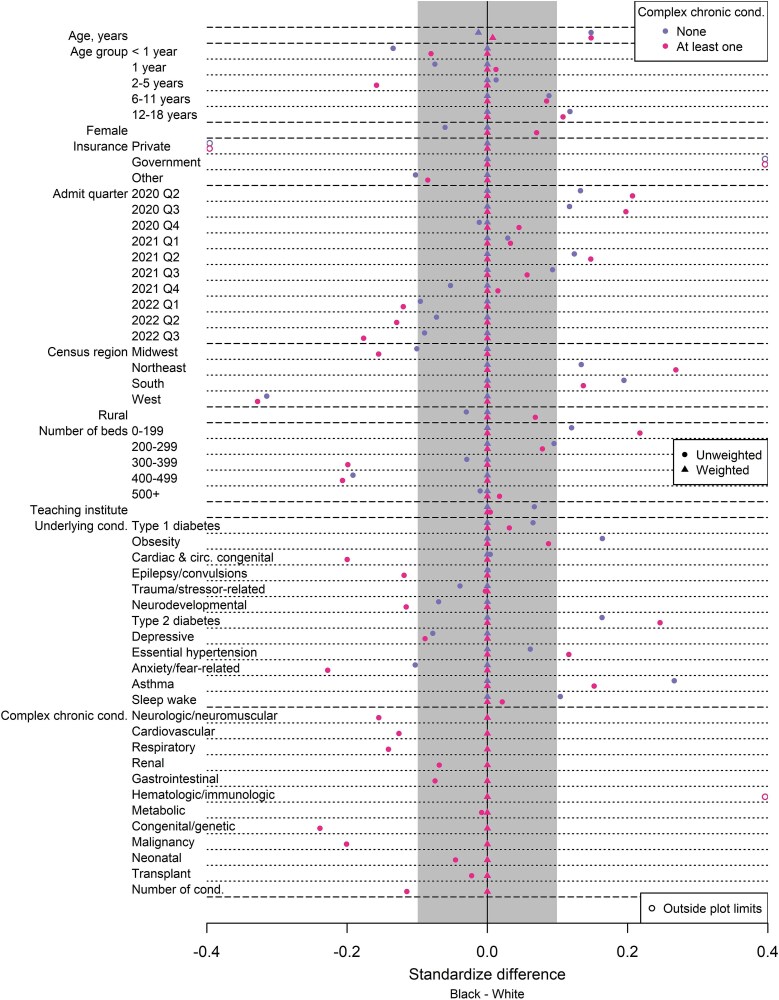
Comparisons of Black and White Inpatients with COVID-19 with Respect to Baseline Characteristics before and after Weighting among Subgroups of Complex Chronic Conditions. Legend: Before weighting, Black and White groups were not comparable because several factors were severely imbalanced as indicated by standardized differences greater than 0.1 in absolute value (ie, outside the grey-shaded region). After propensity score weighting, Black and White inpatient groups were comparable because both had the same average value for every factor as indicated by standardized differences of 0. Cond.: condition.

Similar patterns of differences between racial and ethnic groups were observed within subgroups of effect modifiers. For example, Figure 1 shows standardized differences between Black and White patients for all confounding factors within subgroups of CCCs (ie, none versus at least one), and several confounders were imbalanced (ie, absolute values of standardized differences greater than 0.1). However, after applying overlap weights, the two groups were the same on average for all the confounders. This approach was applied separately for the analysis of each effect modifier so racial and ethnic groups were comparable within each subgroup of an effect modifier.

### Effect Modification of Disparities Comparing Black and White Inpatients

**Figure 2:**
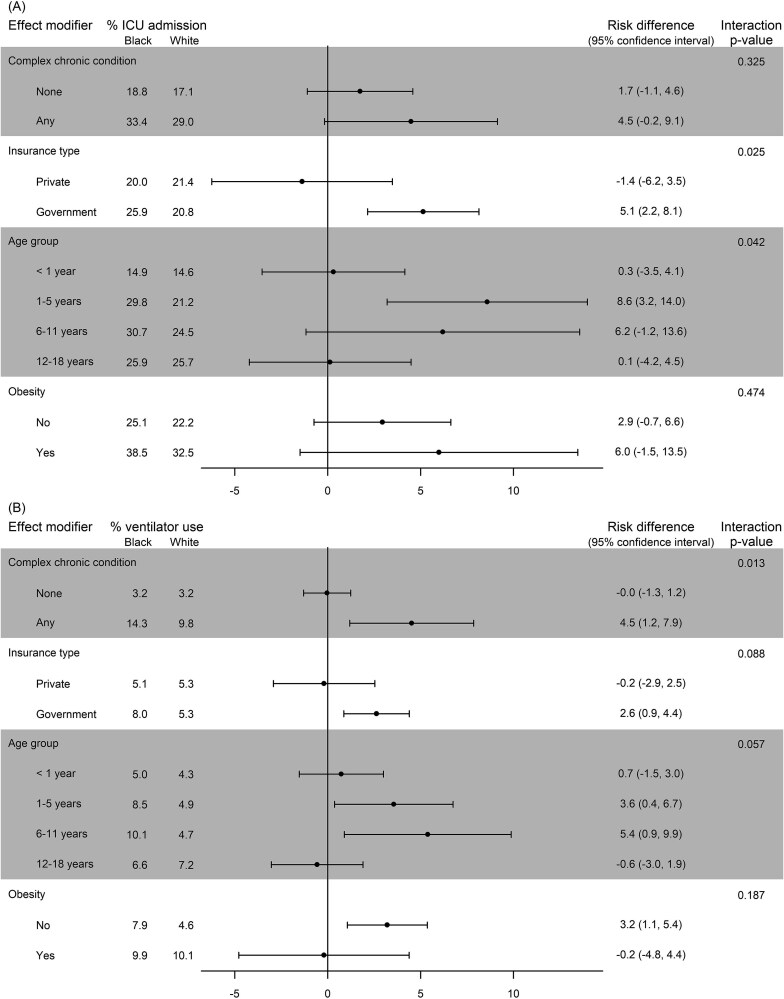
Adjusted Comparisons of Black and White COVID-19 Severity Outcomes within Subgroups of Potential Effect Modifiers. Legend: For the outcomes of (A) intensive care unit (ICU) admission and (B) ventilator use, comparisons are presented as risk differences in percentage points. The interaction *P*-value tests for heterogeneity of disparities; a statistically significant result suggests effect modification is present. Results adjusted for patient and provider characteristics.

Within each subgroup of a potential effect modifier, adjusted comparisons between Black and White inpatients for COVID-19 severity outcomes are reported in Figure 2 (see Table S1 for unadjusted results). Among children with a CCC, 14.3% of Black inpatients required a ventilator compared to 9.8% of White inpatients for an RD of 4.5 percentage points (95% CI, 1.2, 7.9), whereas among children without a CCC, 3.2% of both Black and White inpatients required a ventilator (RD = 0.0, 95% CI, −1.3, 1.2), and the comparison of these two RDs was statistically significant (interaction *P*=.013). For the outcome of ICU admission, 33.4% of Black inpatients with a CCC were admitted to the ICU compared to 29.0% of White inpatients (RD = 4.5, 95% CI, −0.2, 9.1), and 18.8% of Black inpatients without a CCC were admitted to the ICU compared to 17.1% of White inpatients (RD = 1.7, 95% CI, −1.1, 4.8), but the comparison of these disparities was not statistically significant (interaction *P* = .325).

Among children with government insurance, 25.9% of Black inpatients were admitted to the ICU compared to 20.8% of White inpatients for an RD of 5.1 percentage points (95% CI, 2.2, 8.1). In contrast, among children with private insurance, Black and White inpatients had comparable ICU admission rates of 20.0% and 21.4%, respectively (RD = −1.4, 95% CI, −6.2, 3.5). The comparison of these disparities was statistically significant (interaction *P* = .025). A similar pattern was observed for Black-versus-White disparities in ventilator use, with larger disparities among inpatients with government insurance (RD = 2.6, 95% CI, 0.9, 4.4) and no disparity among inpatients with private insurance (RD = −0.2, 95% CI, −2.9, 2.5); however, the comparison of these disparities was not statistically significant (interaction *P* = .088).

Disparities were largest among children 1 to 5 years old (RD = 8.2, 95% CI, 3.2, 14.0) and 6 to 11 years old (RD = 6.2, 95% CI: −1.2, 13.6). In contrast, ICU admission rates were similar between Black and White inpatients among children less than 1 year old (RD = 0.3, 95% CI, −3.5, 4.1) and 12- to 18-year-olds (RD = 0.1, 95% CI, −4.2, 4.5). The comparison of these disparities was statistically significant (interaction *P* = .042). A similar pattern of Black-versus-White disparities was observed for ventilator use across these age groups, but the result was not statistically significant (interaction *P* = .057).

Patients with obesity had higher rates of ICU admission and ventilator use, but obesity did not modify Black-versus-White disparities. Additionally, vaccine eligibility was not an effect modifier of disparities (see online supplementary materials).

### Effect Modification of Disparities Comparing Hispanic and White Inpatients

**Figure 3:**
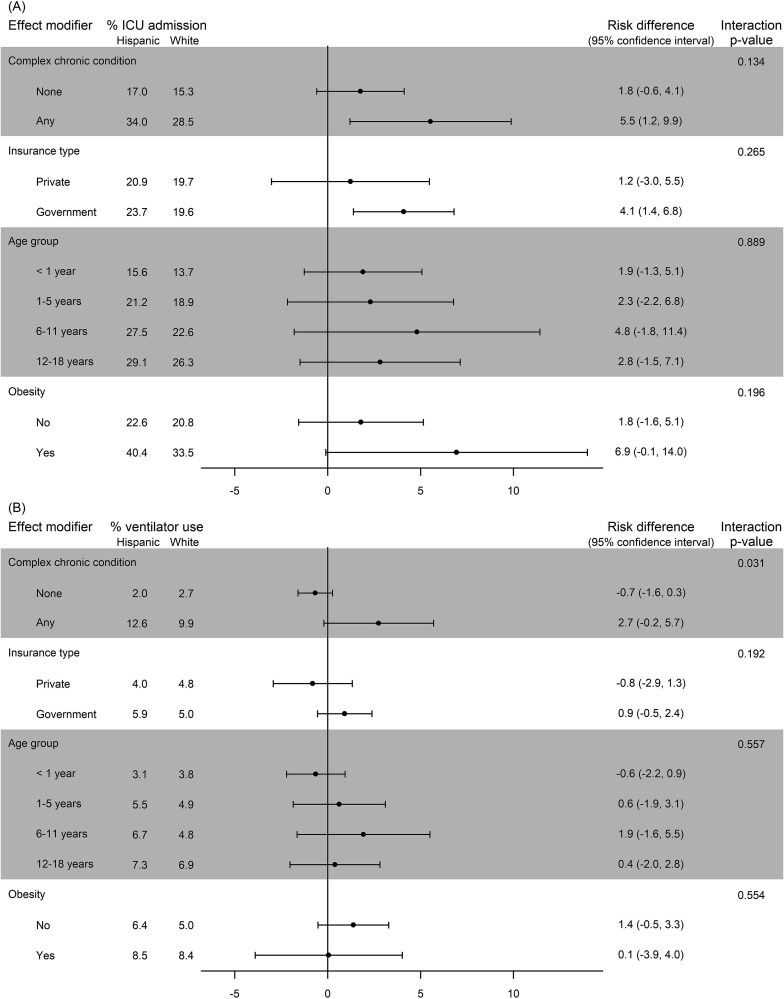
Adjusted Comparisons of Hispanic and White COVID-19 Severity Outcomes within Subgroups of Potential Effect Modifiers. Legend: For the outcomes of (A) intensive care unit (ICU) admission and (B) ventilator use, comparisons are presented as risk differences in percentage points. The interaction *P*-value tests for heterogeneity of disparities; a statistically significant result suggests effect modification is present. Results adjusted for patient and provider characteristics.

Adjusted comparisons of COVID-19 severity outcomes between Hispanic and White inpatients within each subgroup of an effect modifier are reported in Figure 3 (see Table S2 for unadjusted results). Among children with a CCC, 12.6% of Hispanic inpatients required a ventilator compared to 9.9% of White inpatients for an RD of 2.7 percentage points (95% CI, −0.2, 5.7). In contrast, among children with no CCCs, 2.0% of Hispanic inpatients used a ventilator compared to 2.7% of White inpatients (RD = −0.7, 95% CI, −1.6, 0.3). The comparison of these disparities was statistically significant (interaction *P* = .031). A similar but not statistically significant pattern was observed in Hispanic-versus-White disparities of ICU admissions (interaction *P* = .134). Among children with at least one CCC, 34.0% of Hispanic inpatients were admitted to the ICU compared to 28.5% of White inpatients for an RD of 5.5 percentage points (95% CI, 1.2, 9.9), whereas among children with no CCC the rate of ICU admission was only slightly higher in Hispanic inpatients compared to White inpatients (RD = 1.8, 95% CI, −0.6, 4.1).

Insurance type was not an effect modifier of Hispanic-versus-White disparities for both outcomes of COVID-19 severity. Among children with government insurance, 23.7% of Hispanic inpatients were admitted to the ICU compared to 19.6% of White inpatients for an RD of 4.1 percentage points (95% CI, 1.4, 6.8), and among children with private insurance, 20.9% of Hispanic inpatients were admitted to the ICU compared to 19.7% of White inpatients for an RD of 1.2 percentage points (95% CI, −3.0, 5.5). The comparison of these disparities was not statistically significant (interaction *P* = .265). Rates of ventilator use were similar between Hispanic and White inpatients with government insurance (RD = 0.9, 95% CI, −0.5, 2.4) and private insurance (RD = -0.8, 95% CI, −2.9, 1.3, interaction *P* = .192).

For ICU admission, the largest disparity was among 6- to 11-year-olds (RD = 4.8, 95% CI, −1.8, 11.4). Age was not an effect modifier of Hispanic-versus-White disparities for either outcome. Similarly, obesity was not an effect modifier of Hispanic-versus-White disparities, though patients with obesity had higher rates of ICU admission and ventilator use. Additionally, vaccine eligibility was not an effect modifier of Hispanic-versus-White disparities (see online supplementary materials).

## DISCUSSION

This study examined differences in the burden of COVID-19 severity experienced by Black and Hispanic pediatric patients relative to White patients and identified certain subgroups that experienced the largest disparities. In examining effect modifiers of disparities, this work expands on traditional disparities research that identifies differences in health between groups of people who are more socially advantaged and those who are less socially advantaged.^24^ We emphasize that race and ethnicity are social constructs and do not ascribe a biological explanation for the observed differences in COVID-19 severity.^25^ Within the USA, disparities in both health and social advantage have occurred within historical discriminatory practices that affect healthcare and other aspects of social life.^26^

In more detail, we found that having at least one CCC was an effect modifier of Black-versus-White disparities and Hispanic-versus-White disparities. In other words, among inpatients with complex comorbidities, Black and Hispanic children had more severe COVID-19 illness than White children on average, yet among children without complex comorbidities Black and Hispanic children had a similar burden of COVID-19 severity compared to White children. This result affirms a prior finding that Black-versus-White disparities in hospitalization rates were largest among children with CCCs relative to children without CCCs.^7^ The main distinction of this work is focusing on inpatient encounters using additional COVID-19 severity indicators and extending this result to Hispanic-versus-White disparities. Several studies have reported associations between comorbidities and COVID-19 severity as well as other viral respiratory tract infections such as respiratory syncytial virus (RSV) and influenza.^27-29^ A modeling study suggested that interventions equalizing comorbidities would be most effective to close the gap in race-based inequities for influenza hospitalizations.^30^ In the context of our results on COVID-19, we showed that Black inpatients were much more likely to have CCCs and that CCCs were associated with more severe outcomes; however, disparities were largest among children with CCCs, suggesting equalizing comorbidities may not be enough to eliminate these disparities.

Insurance type was also an effect modifier of Black-versus-White disparities in COVID-19 severity. That is, among inpatients with government insurance, Black children had more severe COVID-19 burden than White children, whereas among inpatients with private insurance, Black and White children had comparable COVID-19 severity. Insurance status can be used as a proxy measure of socioeconomic status, but this one metric does not capture the entirety of patients’ resources.^31^ Other aspects or measures may better capture social determinants of health, for example, more detailed patient and family data (eg, English-language proficiency, housing information, parental education level, food insecurity, and parental employment) or neighborhood-level summaries (eg, area deprivation index,^32^ child opportunity index,^33^ and census tract data). Many of these measures have been linked to pediatric COVID-19 severity^34-38^ but were unavailable within the database, which is a major limitation of this study. Future work should consider these measures as potential effect modifiers of racial and ethnic disparities.

The American Academy of Pediatrics’ Equity Agenda promotes health equity via identification and elimination of racial and ethnic inequity.^39^^,^^40^ Our analysis of factors impacting COVID-19 severity in children homes in on the identification of disparities and can aid in eliminating inequity. Just as it is important to identify subgroups at highest risk for infections to promote targeted preventative interventions (ie, vaccines), it is also important to identify subpopulations experiencing the largest disparities in disease burden to find ways to promote equity within such groups. Research on interventions to reduce health disparities suggests there is no one-size-fits-all solution, and thus interventions need to be multifaceted and tailored to specific communities.^34-36^ We suggest that part of the tailoring process includes identifying populations that would benefit most from such interventions and that assessing effect modification can help identify the medical factors (eg, CCCs) and social determinants of health (eg, insurance status) that contribute to existing health disparities. Although the brunt of the COVID-19 pandemic has passed, similar associations have been observed among other respiratory illnesses, suggesting that disease-agnostic structural interventions may help reduce disparities in pediatric respiratory viral infections via improving access to care and prevention strategies for more vulnerable communities.^41^

This study has limitations because of the observational study design, which is inherent in empirical examinations of racial disparities. We attempted to adjust for observed confounding factors using a novel methodology that ensured racial groups were comparable within subgroups of effect modifiers;^18^^,^^21^ however, certain factors could not be measured. In addition to the aforementioned measures of social determinants of health, vaccination status could not be reliably obtained in our data, and prior studies report racial and ethnic disparities in COVID-19 vaccine uptake,^42^^,^^43^ making vaccination status a known but unascertained confounding factor. To address this major limitation, we performed a sensitivity analysis that indirectly assessed the potential for confounding by unknown vaccine status by considering age specific eligibility criteria for COVID-19 vaccination (see the online supplementary materials). However, this sensitivity analysis was limited as well because the results cannot be solely attributable to vaccine availability and may coincide with other factors (eg, differential COVID-19 presentation of coronavirus variants^44^ and/or changing public health policies). Lastly, the racial and ethnic categorizations used in this study are similar to those used in prior work, but these categorizations have limitations, especially among Hispanic patients, for whom race data may be misclassified or ambiguous (eg, “other” or “unknown”).^45^ We acknowledge that the Hispanic group in this study was racially heterogeneous but also view this group as a social construct that has been historically disadvantaged relative to the non-Hispanic White population.

## CONCLUSIONS

In this study, complex comorbidities and insurance status impacted racial and ethnic disparities in COVID-19 severity among hospitalized children. The results of this study are important for a better understanding of racial and ethnic disparities in COVID-19 severity and other potential health outcomes. Further investigation is needed to determine strategies to reduce these disparities.

## Supplementary Material

Watson_revision_supplement_JPIDS_piaf092

## References

[1] Moreira A, Chorath K, Rajasekaran K, Burmeister F, Ahmed M, Moreira A. Demographic predictors of hospitalization and mortality in US children with COVID-19. *Eur J Pediatr* 2021;180:1659–1663. 10.1007/s00431-021-03955-x 33474580 PMC7817069

[2] Woodruff RC, Campbell AP, Taylor CA et al. Risk factors for severe COVID-19 in children. *Pediatrics* 2022;149:2021053418. 10.1542/peds.2021-053418 PMC9213563 34935038

[3] Kim L, Whitaker M, O'Halloran A et al. Hospitalization rates and characteristics of children aged <18 years hospitalized with laboratory-confirmed COVID-19 - COVID-NET, 14 states, March 1-July 25, 2020. *MMWR* 2020;69:1081–1088. 10.15585/mmwr.mm6932e3 32790664 PMC7440125

[4] Kompaniyets L, Agathis NT, Nelson JM et al. Underlying medical conditions associated with severe COVID-19 illness among children. *JAMA Netw Open* 2021;4:e2111182. 10.1001/jamanetworkopen.2021.11182 34097050 PMC8185607

[5] Aparicio C, Willis ZI, Nakamura MM et al. Risk factors for pediatric critical COVID-19: a systematic review and meta-analysis. *J Pediatric Infect Dis Soc* 2024;13:352–362. 10.1093/jpids/piae052 38780125 PMC11519042

[6] Yaeger JP, Alio AP, Fiscella K. Addressing child health equity through clinical decision-making. *Pediatrics* 2022;149:2021053698. 10.1542/peds.2021-053698 35102415

[7] Watson D, Spaulding A, Norton L. Effect modification of racial differences in pediatric COVID-19 inpatient admissions in a large healthcare database. *Pediatr Infect Dis J* 2023;42:594–600. 10.1097/INF.0000000000003930 37171975 PMC10289073

[8] PREMIER . Premier Healthcare Database: data that informs and performs. 2020. Accessed January 20, 2023. https://products.premierinc.com/downloads/PremierHealthcareDatabaseWhitepaper.pdf

[9] PREMIER . Research needs robust data: the CDC utilizes the PINC AI™ Healthcare Database. 2022. Accessed January 20, 2023. https://premierinc.com/newsroom/blog/research-needs-robust-data-the-cdc-utilizes-the-pinc-ai-healthcare-database

[10] Feudtner C, Feinstein JA, Zhong W, Hall M, Dai D. Pediatric complex chronic conditions classification system version 2: updated for ICD-10 and complex medical technology dependence and transplantation. *BMC Peds* 2014;14:1–7. 10.1186/1471-2431-14-199 PMC4134331 25102958

[11] Gelman A, Hill J, Vehtari A. Ch. 16, Design and sample size decisions. In:*Regression and Other Stories*. C.U. Press, 2021.

[12] AHRQ . Chronic Condition Indicator Refined (CCIR) for ICD-10-CM. 2024. Accessed January 5, 2025. https://hcup-us.ahrq.gov/toolssoftware/chronic_icd10/chronic_icd10.jsp

[13] AHRQ . Clinical Classifications Software Refined (CCSR) for ICD-10-CM diagnose*s*. 2024. Accessed January 5, 2025. https://hcup-us.ahrq.gov/toolssoftware/ccsr/dxccsr.jsp

[14] Feudtner C, Christakis DA, Connell FA. Pediatric deaths attributable to complex chronic conditions: a population-based study of Washington State, 1980-1997. *Pediatrics* 2000;106:205–209. 10.1542/peds.106.S1.205 10888693

[15] Rosenbaum PR, Rubin DB. The central role of the propensity score in observational studies for causal effects. *Biometrika* 1983;70:41–55. 10.1093/biomet/70.1.41

[16] Lê Cook B, McGuire TG, Lock K, Zaslavsky AM. Comparing methods of racial and ethnic disparities measurement across different settings of mental health care. *Health Serv Res* 2010;45:825–847. 10.1111/j.1475-6773.2010.01100.x 20337739 PMC2875762

[17] Ye Y, Bond JC, Schmidt LA, Mulia N, Tam TW. Toward a better understanding of when to apply propensity scoring: a comparison with conventional regression in ethnic disparities research. *Ann Epidemiol* 2012;22:691–697. 10.1016/j.annepidem.2012.07.008 22902041 PMC3494414

[18] Li F, Li F. Using propensity scores for racial disparities analysis. *Obs Stud* 2023;9:59–68. 10.1353/obs.2023.0005

[19] Li F . Overlap weighting. In:*Handbook of Matching and Weighting Adjustments for Causal Inference*, pp. 263–282. Chapman and Hall/CRC, 2023 https://doi.org/10.1201/9781003102670-14.

[20] Green KM, Stuart EA. Examining moderation analyses in propensity score methods: application to depression and substance use. *J Consult Clin Psychol* 2014;82:773–783. 10.1037/a0036515 24731233 PMC4172552

[21] Yang S, Lorenzi E, Papadogeorgou G, Wojdyla DM, Li F, Thomas LE. Propensity score weighting for causal subgroup analysis. *Stat Med* 2021;40:4294–4309. 10.1002/sim.9029 33982316 PMC8360075

[22] Austin PC, Stuart EA. Moving towards best practice when using inverse probability of treatment weighting (IPTW) using the propensity score to estimate causal treatment effects in observational studies. *Stat Med* 2015;34:3661–3679. 10.1002/sim.6607 26238958 PMC4626409

[23] Rubin DB . The design versus the analysis of observational studies for causal effects: parallels with the design of randomized trials. *Stat Med* 2007;26:20–36. 10.1002/sim.2739 17072897

[24] Braveman P . Health disparities and health equity: concepts and measurement. *Annu Rev Public Health* 2006;27:167–194. 10.1146/annurev.publhealth.27.021405.102103 16533114

[25] Smedley A, Smedley BD. Race as biology is fiction, racism as a social problem is real: anthropological and historical perspectives on the social construction of race. *Am Psychol* 2005;60:16–26. 10.1037/0003-066X.60.1.16 15641918

[26] Nelson A . Unequal treatment: confronting racial and ethnic disparities in health care. *J Natl Med Assoc* 2002;94:666–668. 12152921 PMC2594273

[27] Curns AT, Rha B, Lively JY et al. Respiratory syncytial virus-associated hospitalizations among children <5 years old: 2016 to 2020. *Pediatrics* 2024;153:2023062574. 10.1542/peds.2023-062574 38298053

[28] Brenes-Chacon H, Eisner M, Acero-Bedoya S, Ramilo O, Mejias A. Age-specific predictors of disease severity in children with respiratory syncytial virus infection beyond infancy and through the first 5 years of age. *Pediatr Allergy Immunol* 2024;35:e14083. 10.1111/pai.14083 38363050

[29] Gill PJ, Ashdown HF, Wang K et al. Identification of children at risk of influenza-related complications in primary and ambulatory care: a systematic review and meta-analysis. *Lancet Respir Med* 2015;3:139–149. 10.1016/S2213-2600(14)70252-8 25481379

[30] Stafford E, Dimitrov D, Trinidad SB, Matrajt L. Closing the gap in race-based inequities for seasonal influenza hospitalizations: a modeling study. *Clin Infect Dis* 2024;81:478–487. 10.1093/cid/ciae564 PMC12497953 39560376

[31] Monuteaux MC, Du M, Neuman MI. Evaluation of insurance type as a proxy for socioeconomic status in the Pediatric emergency department: a pilot study. *Ann Intern Med* 2024;83:562–567. 10.1016/j.annemergmed.2023.12.013 38244029

[32] Kind AJH, Buckingham WR. Making neighborhood-disadvantage metrics accessible - the Neighborhood Atlas. *N Engl J Med* 2018;378:2456–2458. 10.1056/NEJMp1802313 29949490 PMC6051533

[33] Acevedo-Garcia D, McArdle N, Hardy EF et al. The child opportunity index: improving collaboration between community development and public health. *Health Aff* 2014;33:1948–1957. 10.1377/hlthaff.2014.0679 25367989

[34] Madhav KC, Oral E, Straif-Bourgeois S, Rung AL, Peters ES. The effect of area deprivation on COVID-19 risk in Louisiana. *PLoS One* 2020;15:e0243028. 10.1371/journal.pone.0243028 33270701 PMC7714106

[35] Shah RM, Parzen-Johnson S, Sun S, Patel SJ. Childhood opportunity index and vaccine uptake in pediatric COVID-19 hospitalizations. *Vaccine* 2025;48:126734. 10.1016/j.vaccine.2025.126734 39823851

[36] Tyler S, Abuogi L, Vannoni V et al. Mixed methods evaluation of the impact of the COVID-19 pandemic on immigrant families. *Hisp Health Care Int* 2024;22:11–24. 10.1177/15404153231214707 37981744

[37] Lang S, Silveira L, Smith C, Abuogi L, DeCamp LR. Variation over time in child and neighborhood characteristics associated with COVID-19. *Health Equity* 2023;7:676–684. 10.1089/heq.2022.0213 37908402 PMC10615088

[38] Graff K, Choi YJ, Silveira L et al. Lessons learned for preventing health disparities in future pandemics: the role of social vulnerabilities among children diagnosed with severe COVID-19 early in the pandemic. *AIMS Public Health* 2025;12:124–136. 10.3934/publichealth.2025009 40248413 PMC11999811

[39] American Academy of Pediatrics . AAP equity agenda. 2025. Accessed January 5, 2025. https://www.aap.org/en/about-the-aap/american-academy-of-pediatrics-equity-and-inclusion-efforts/aap-equity-agenda/

[40] American Academy of Pediatrics . AAP Diversity and Inclusion Statement. *Pediatrics* 2018;**141**:e20180193. 10.1542/peds.2018-0193 29555690

[41] Brown AF, Ma GX, Miranda J et al. Structural interventions to reduce and eliminate health disparities. *Am J Public Health* 2019;109:S72–S78. 10.2105/AJPH.2018.304844 30699019 PMC6356131

[42] Na L, Banks S, Wang PP. Racial and ethnic disparities in COVID-19 vaccine uptake: a mediation framework. *Vaccine* 2023;41:2404–2411. 10.1016/j.vaccine.2023.02.079 36894396 PMC9974364

[43] Nguyen LH, Joshi AD, Drew DA et al. Self-reported COVID-19 vaccine hesitancy and uptake among participants from different racial and ethnic groups in the United States and United Kingdom. *Nat Comms* 2022;13:636. 10.1038/s41467-022-28200-3 PMC8807721 35105869

[44] Lefchak B, Nickel A, Lammers S, Watson D, Hester GZ, Bergmann KR. Analysis of COVID-19–related croup and SARS-CoV-2 variant predominance in the US. *JAMA Net Open* 2022;5:e2220060–e2220060. 10.1001/jamanetworkopen.2022.20060 PMC9250054 35796213

[45] Allen VC Jr, Lachance C, Rios-Ellis B, Kaphingst KA. Issues in the assessment of "race" among Latinos: implications for research and policy. *Hisp J Behav Sci* 2011;33:411–424. 10.1177/0739986311422880 23239903 PMC3519364

