# Supplementary material for: Heterogeneity in Racial and Ethnic Disparities in COVID-19 Severity Among Pediatric Inpatients in a National Healthcare Database
Source: J Pediatric Infect Dis Soc. 2025 Oct 8;14(10):piaf092. doi: 10.1093/jpids/piaf092 (PMC12570028; doi:10.1093/jpids/piaf092)
Supplement: Watson_revision_supplement_JPIDS_piaf092 [file watson_revision_supplement_jpids_piaf092.docx]

**Supplemental Figure 1.** Flowchart of cohort inclusion from Premier Healthcare Database inpatient encounters <19 years of age with diagnosis of COVID-19, January 2020 through September 2022.


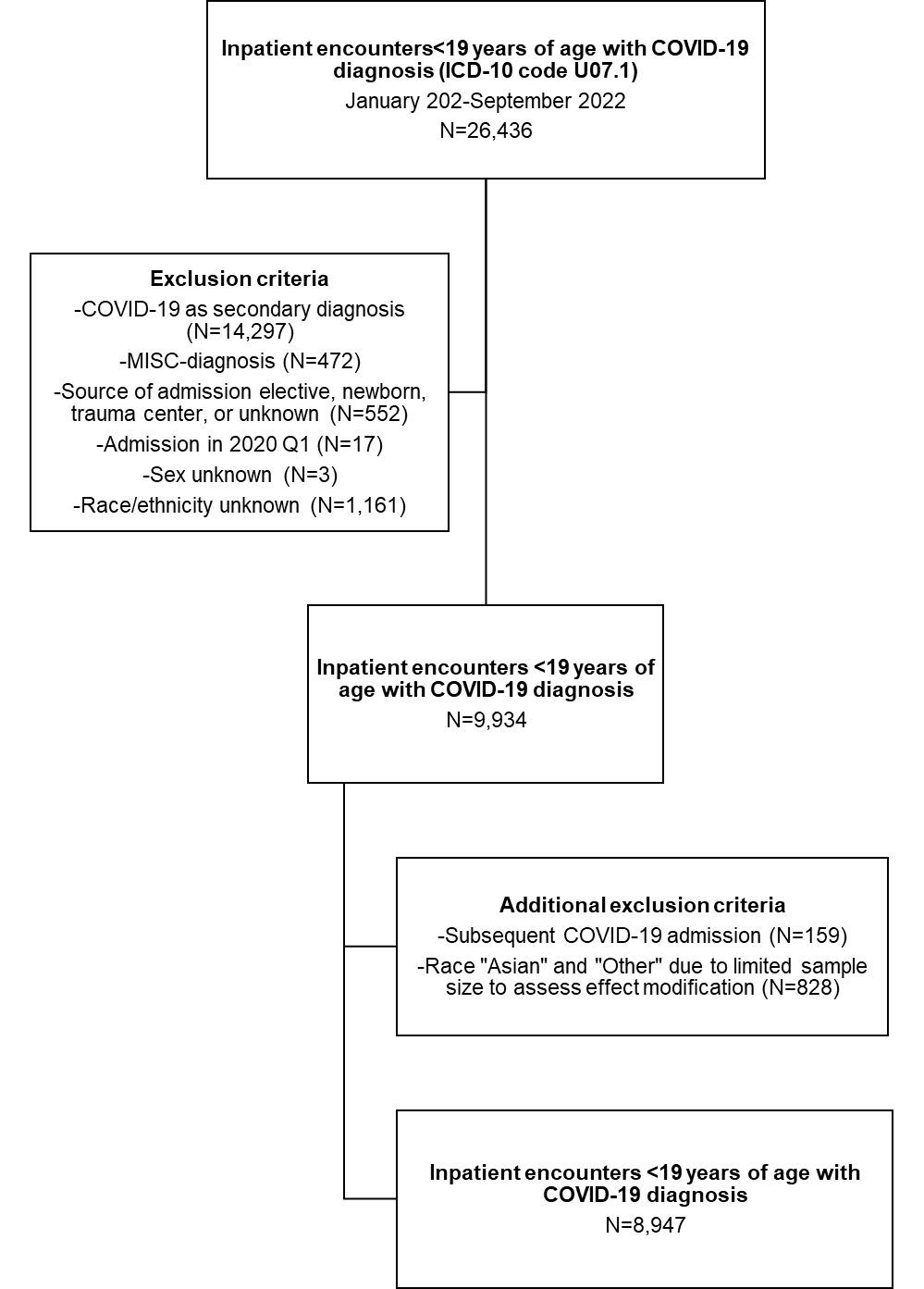


**Supplemental Table 1.** Frequencies and comparisons of Black and White COVID-19 severity outcomes within subgroups of effect modifiers.

|  |  |  | **Percent (n/N)** | | **Difference** | **Interaction** |
| --- | --- | --- | --- | --- | --- | --- |
| **Outcome** | **Effect Modifier** | **Subgroup** | **Black** | **White** | **(95% conf. int.)** | **P-value** |
| ICU admission | Comp. chr. cond. |  |  |  |  | 0.691 |
|  |  | None | 19.8 (239/1210) | 16.3 (430/2635) | 3.4 (0.8, 6.1) |  |
|  |  | Any | 31.2 (294/943) | 28.7 (351/1223) | 2.5 (-1.4, 6.4) |  |
|  | Insurance type |  |  |  |  | 0.008 |
|  |  | Private | 19.2 (76/395) | 20.3 (344/1696) | -1.0 (-5.4, 3.3) |  |
|  |  | Government | 26.2 (438/1669) | 20.3 (389/1913) | 5.9 (3.1, 8.7) |  |
|  | Age group |  |  |  |  | 0.006 |
|  |  | < 1 year | 14.7 (79/536) | 15.5 (193/1247) | -0.7 (-4.3, 2.9) |  |
|  |  | 1-5 years | 29.0 (136/469) | 20.3 (193/952) | 8.7 (3.9, 13.6) |  |
|  |  | 6-11 years | 29.6 (99/334) | 21.2 (96/453) | 8.4 (2.3, 14.6) |  |
|  |  | 12-18 years | 26.9 (219/814) | 24.8 (299/1206) | 2.1 (-1.8, 6.0) |  |
|  | Obesity |  |  |  |  | 0.526 |
|  |  | No | 22.1 (395/1791) | 18.6 (631/3395) | 3.5 (1.1, 5.8) |  |
|  |  | Yes | 38.1 (138/362) | 32.4 (150/463) | 5.7 (-0.8, 12.3) |  |
| Ventilator use | Comp. chr. cond. |  |  |  |  | 0.299 |
|  |  | None | 3.5 (42/1210) | 3.2 (84/2635) | 0.3 (-0.9, 1.5) |  |
|  |  | Any | 12.0 (113/943) | 10.1 (124/1223) | 1.8 (-0.8, 4.5) |  |
|  | Insurance type |  |  |  |  | 0.006 |
|  |  | Private | 4.3 (17/395) | 5.6 (95/1696) | -1.3 (-3.6, 1.0) |  |
|  |  | Government | 8.0 (133/1669) | 5.3 (102/1913) | 2.6 (1.0, 4.3) |  |
|  | Age group |  |  |  |  | 0.068 |
|  |  | < 1 year | 5.4 (29/536) | 4.2 (52/1247) | 1.2 (-1.0, 3.5) |  |
|  |  | 1-5 years | 7.9 (37/469) | 4.7 (45/952) | 3.2 (0.4, 5.9) |  |
|  |  | 6-11 years | 9.0 (30/334) | 4.2 (19/453) | 4.8 (1.2, 8.4) |  |
|  |  | 12-18 years | 7.2 (59/814) | 7.6 (92/1206) | -0.4 (-2.7, 1.9) |  |
|  | Obesity |  |  |  |  | 0.426 |
|  |  | No | 6.6 (118/1791) | 4.7 (161/3395) | 1.8 (0.5, 3.2) |  |
|  |  | Yes | 10.2 (37/362) | 10.2 (47/463) | 0.1 (-4.1, 4.2) |  |

Abbreviations: conf. Int., confidence interval; ICU, intensive care unit; comp. chr. cond., complex chronic condition.

**Supplemental Table 2.** Frequencies and comparisons of Hispanic and White COVID-19 severity outcomes within subgroups of effect modifiers.

|  |  |  | **Percent (n/N)** | | **Difference** | **Interaction** |
| --- | --- | --- | --- | --- | --- | --- |
| **Outcome** | **Effect Modifier** | **Subgroup** | **Hispanic** | **White** | **(95% conf. int.)** | **P-value** |
| ICU admission | Comp. chr. cond. |  |  |  |  | 0.098 |
|  |  | None | 17.5 (346/1972) | 16.3 (430/2635) | 1.2 (-1.0, 3.4) |  |
|  |  | Any | 33.7 (325/964) | 28.7 (351/1223) | 5.0 (1.1, 8.9) |  |
|  | Insurance type |  |  |  |  | 0.492 |
|  |  | Private | 21.5 (104/483) | 20.3 (344/1696) | 1.2 (-2.9, 5.4) |  |
|  |  | Government | 23.3 (509/2186) | 20.3 (389/1913) | 2.9 (0.4, 5.5) |  |
|  | Age group |  |  |  |  | 0.021 |
|  |  | < 1 year | 15.0 (152/1010) | 15.5 (193/1247) | -0.4 (-3.4, 2.6) |  |
|  |  | 1-5 years | 21.5 (148/687) | 20.3 (193/952) | 1.3 (-2.7, 5.3) |  |
|  |  | 6-11 years | 29.6 (110/371) | 21.2 (96/453) | 8.5 (2.5, 14.4) |  |
|  |  | 12-18 years | 30.1 (261/868) | 24.8 (299/1206) | 5.3 (1.4, 9.2) |  |
|  | Obesity |  |  |  |  | 0.036 |
|  |  | No | 19.6 (487/2481) | 18.6 (631/3395) | 1.0 (-1.0, 3.1) |  |
|  |  | Yes | 40.4 (184/455) | 32.4 (150/463) | 8.0 (1.8, 14.2) |  |
| Ventilator use | Comp. chr. cond. |  |  |  |  | 0.031 |
|  |  | None | 2.2 (43/1972) | 3.2 (84/2635) | -1.0 (-1.9, -0.1) |  |
|  |  | Any | 12.2 (118/964) | 10.1 (124/1223) | 2.1 (-0.6, 4.8) |  |
|  | Insurance type |  |  |  |  | 0.052 |
|  |  | Private | 3.7 (18/483) | 5.6 (95/1696) | -1.9 (-3.9, 0.1) |  |
|  |  | Government | 5.9 (129/2186) | 5.3 (102/1913) | 0.6 (-0.8, 2.0) |  |
|  | Age group |  |  |  |  | 0.147 |
|  |  | < 1 year | 3.2 (32/1010) | 4.2 (52/1247) | -1.0 (-2.6, 0.5) |  |
|  |  | 1-5 years | 5.7 (39/687) | 4.7 (45/952) | 0.9 (-1.2, 3.1) |  |
|  |  | 6-11 years | 7.0 (26/371) | 4.2 (19/453) | 2.8 (-0.4, 6.0) |  |
|  |  | 12-18 years | 7.4 (64/868) | 7.6 (92/1206) | -0.3 (-2.6, 2.0) |  |
|  | Obesity |  |  |  |  | 0.381 |
|  |  | No | 4.9 (122/2481) | 4.7 (161/3395) | 0.2 (-0.9, 1.3) |  |
|  |  | Yes | 8.6 (39/455) | 10.2 (47/463) | -1.6 (-5.3, 2.2) |  |

Abbreviations: conf. Int., confidence interval; ICU, intensive care unit; comp. chr. cond., complex chronic condition.

**Sensitivity analysis for unknown vaccination status**

**Supplemental Table 3.** Adjusted comparisons of Black-versus-White and Hispanic-versus-White COVID-19 severity outcomes using vaccination eligibility as an effect modifier in order to conduct a sensitivity analysis for unknown vaccination status.

| Comparison | Outcome | Vaccine  eligible | Non-white | White | Risk difference  (95% conf. int.) | Interaction  p-value |
| --- | --- | --- | --- | --- | --- | --- |
| Black vs. White | ICU admission |  |  |  |  | 0.21 |
|  |  | No | 30.2 | 23.5 | 6.8 (2.0, 11.5) |  |
|  |  | Yes | 27.2 | 24.4 | 2.8 (-1.3, 6.9) |  |
|  | Ventilator use |  |  |  |  | 0.58 |
|  |  | No | 6.6 | 4.8 | 1.7 (-0.7, 4.2) |  |
|  |  | Yes | 9.3 | 6.6 | 2.7 (0.2, 5.3) |  |
| Hispanic vs. White | ICU admission |  |  |  |  | 0.26 |
|  |  | No | 26.4 | 21.3 | 5.1 (1.0, 9.1) |  |
|  |  | Yes | 25.9 | 24.1 | 1.8 (-2.1, 5.7) |  |
|  | Ventilator use |  |  |  |  | 0.84 |
|  |  | No | 5.8 | 4.7 | 1.1 (-1.0, 3.2) |  |
|  |  | Yes | 7.2 | 6.4 | 0.8 (-1.5, 3.0) |  |

Abbreviations: conf. Int., confidence interval; ICU, intensive care unit.

Caption: Comparisons are presented as risk differences in percentage points and the interaction p-value tests for heterogeneity of disparities. Results adjusted for patient and provider characteristics.

Definitions, rationale, and results:

Vaccine eligibility was determined based on month of admission with respect to Food and Drug Administration approval and Advisory Committee on Immunization Practices recommendations for first available vaccines for different age groups. Specifically, children 16 years old or older were eligible after December 2020, children 12 to 15 years old were eligible after May 2021, children 5 to 11 years old were eligible after November 2021, and children 1 to 4 years old were eligible after June 2022 (and children less than one were excluded from this analysis). Sample sizes within specific age groups and eligibility timeframes were too small to assess individually.

This sensitivity analysis attempts to assess confounding due to unknown vaccination status, which was unavailable in the Premier Healthcare Database. Vaccination is a known confounding factor because there are known differences in vaccine uptake by racial and ethnic groups and vaccines are known to impact COVID-19 severity. Thus, this sensitivity analysis serves as a comparison of two (age specific) periods when patients were not eligible and eligible for vaccination, with the former group unconfounded with vaccine use (i.e., because it was not available). Thus, the rationale for this sensitivity analysis is to compare differences in disparities (i.e., effect modification) when patients were not eligible and eligible.

Some disparities appeared to differ, but all formal comparisons were not statistically significant (interaction-p > 0.05). Dramatic differences could be attributed to confounding of unobserved vaccination status as well as other possible factors, which makes this sensitivity analysis an imperfect and indirect assessment of unobserved information on vaccination.
